# Petrous bones versus tooth cementum for genetic analysis of aged skeletal remains

**DOI:** 10.1007/s00414-024-03346-5

**Published:** 2024-10-12

**Authors:** Irena Zupanič Pajnič, Tonja Jeromelj, Tamara Leskovar

**Affiliations:** 1 Institute of Forensic Medicine, Faculty of Medicine, University of Ljubljana, Korytkova 2, 1000 Ljubljana, Slovenia; 2 Centre for Interdisciplinary Research in Archaeology, Department of Archaeology, Faculty of Arts, University of Ljubljana, Ljubljana, Slovenia

**Keywords:** Petrous bone, Tooth cementum, Missing person identification, Sampling strategy, Skeletal remains, STR typing

## Abstract

A proper sampling strategy is important to obtain sufficient DNA for successful identification of aged skeletal remains. The petrous bone is the highest DNA-yielding bone in the human body. Because DNA extraction from the petrous bone is very destructive, the demand for other DNA sources is significant. When investigating aged skeletal remains, teeth are usually preserved, and recent studies have shown that DNA in teeth can be best preserved in the dental cementum that surrounds the surface of the tooth root. To extract DNA from the surface of the tooth root, a nondestructive method without grinding was used. Petrous bones and teeth from 60 archaeological adult skeletons were analyzed. The DNA yield, degree of DNA degradation, and STR typing success were compared, and the results showed higher DNA yield and higher amplification success in petrous bones, despite higher degradation of petrous bones’ DNA. The greater success of petrous bones is associated with poorly preserved DNA in a quarter of the teeth analyzed. When teeth with badly preserved DNA were excluded from the statistical analysis, no differences in the success of STR loci amplification were observed even if DNA yield was higher in petrous bones, which can be explained by greater degradation of petrous bones’ DNA. When teeth are well preserved, they can be used for genetically analyzing aged skeletal remains instead of petrous bones, and a rapid nondestructive extraction method can be applied to shorten the identification process and to physically preserve the biological specimen.

**Supplementary Information:**

The online version contains supplementary material available at 10.1007/s00414-024-03346-5.

## Introduction

The pars petrosa of the temporal bone holds substantially greater amounts of endogenous DNA compared to all other bone elements due to its high density [1–7]. Successful ancient genetic analysis has been performed using petrous bone DNA [1, 3, 5, 8, 9], and it has also been used for forensic investigations [4, 10, 11]. Obtaining DNA from the petrous bone is a highly destructive process [5, 12], and so there is a significant need in genetic research for alternative sources of endogenous DNA obtained from other skeletal elements [3, 13]. Besides the petrous bone’s otic capsule, tooth cementum, which covers the outer layer of the tooth roots, was also recently considered a very good source of DNA in investigations of ancient skeletons [1, 5, 14, 15]. Teeth are often collected in forensic analysis in missing person identification cases when skeletonized human remains are found [16, 17]. They are preferred in comparison to bones because of their very low porosity and hard mineral composition, making them less likely to be affected by taphonomy and contamination [18–20]. In addition, sampling them is very practical [21]. Tooth cementum is rich in DNA-containing cells known as cementocytes [14, 22], which are mainly present in the tooth root apex [23]. Cementum apposition continues throughout life [24], and the thickness of cementum increases with age [25]. Because of destructive sampling, the use of the petrous bone in forensic analysis of skeletal remains is not a common practice. In contrast to the petrous bone, the cementum on the tooth root surface is easily accessible, and a nondestructive method can be used for DNA extraction [26].

To explore the usefulness of tooth cementum in comparison to the petrous bone for genetic analysis of aged skeletal remains, 60 archaeological adult skeletons were analyzed, and DNA yield, degree of DNA degradation, and short tandem repeat (STR) amplification success were compared.

It is extremely difficult to acquire a substantial set of aged bones and teeth from forensic cases, and so 60 adult skeletons from two archaeological sites served as models of poorly preserved skeletal remains in this study. Three parameters (DNA yield, degradation index, and STR amplification success) were used to compare petrous bones and tooth cementum and determine the usefulness of dental cementum for genetic identification of aged skeletal remains.

## Materials and methods

### Selection of bone and tooth samples

Thirty adult skeletons originating from the Črnomelj archeological site and another 30 skeletons of adults from the Ljubljana (Vrazov trg—Vraz Square) archeological site were analyzed. To ascertain DNA preservation in petrous bones and tooth cementum, skeletons that had preserved canines and petrous bones were used for the investigation. Skeletons were excavated from modern-era Christian cemeteries that were active between the thirteenth and nineteenth century. Archeological work was performed in Črnomelj in 2019 and 453 graves were discovered, and archeological excavations in Ljubljana were carried out in 2023 and 206 graves were discovered. Most of the skeletons recovered from both archaeological sites were well preserved.

Elimination database samples were genetically typed to exclude the possibility of contamination, and the persons included in the elimination database provided informed consent. Permission for sampling the skeletons from the archaeological sites in Črnomelj and Ljubljana was obtained from the Metlika Museum and from Ljubljana Museum and Galleries (MGML). The research was approved by the Medical Ethics Committee of the Republic of Slovenia (0120–345/2023/6 and 0120–308/2024–2711-3).

### Extraction of DNA

Temporal bones, including petrous bones, and canines were collected from the skeletons. Sampling of petrous bones was carried out following Pinhasi’s method [5], and bone powdering and extraction following [27]. Because cementum was determined to be the part of the tooth where DNA is best preserved [14, 22], DNA from the surface of the tooth root was extracted using a nondestructive method. After chemical cleaning with bleach, water, and ethanol, and UV irradiation using BLX-Multichannel BioLink DNA Crosslinker (Vilber, Collégien, France), the entire tooth was submerged in 10 ml of 0.5 M ethylene diamine tetra acetic acid (EDTA), pH 8 (Promega, Madison, USA), mixing on the Thermomixer comfort (Eppendorf, Hamburg, Germany) at 750 rpm overnight at 37 °C. After centrifugation, the EDTA was discarded and washing with 10 ml of sterile bi-distilled water (Sigma-Aldrich) was carried out. The water was discarded after centrifugation, and lysis of demineralized tooth root tissue was performed with 200 µl of buffer G2 and 120 µl of proteinase K, both supplied with the EZ1 & EZ2 DNA Investigator kit (Qiagen, Hilden, Germany), and 40 µl of 1 M DTT (Promega) for 2 h at 56 °C in the Thermomixer comfort (Eppendorf) at 750 rpm. After centrifugation, whole supernatant was transferred to the EZ1 sample tube, and DNA was purified in an automated DNA purification EZ1 Advanced XL machine (Qiagen), choosing trace protocol, TE buffer, and 50 μl elution volume. The EZ1 & EZ2 DNA Investigator kit (Qiagen) was also utilized for extraction of DNA from buccal swab samples from personnel for the elimination database following the instructions provided by the manufacturer [28], and Biorobot EZ1 (Qiagen) was used for automated DNA purification.

Because DNA extracted from aged skeletal remains is prone to modern DNA contamination, specific measures were applied to monitor and prevent contamination and to verify the validity of genetic profiles acquired from aged skeletons [29–31]. A dedicated room and equipment used only for processing aged tooth and bone samples was used [27]. To build the elimination database, everyone that participated in exhumation and in anthropological and genetic analyses supplied buccal swabs. The elimination database genetic profiles were compared to the profiles obtained from the bones and teeth. Extraction negative controls (ENC) were included in each batch of extraction for contamination monitoring. The degradation index was measured using quantitative PCR to determine the degree of DNA degradation. A high measure of the degradation index was used as an authenticity criterion for ancient DNA. Endogenous DNA was also confirmed through identical genetic profiles obtained from samples of teeth and the petrous bone from the same skeleton.

### Quantitative PCR for DNA quantification

Quantitative PCR (qPCR) using the PowerQuant System (Promega) was performed to measure DNA quantity and DNA quality through targeting the short Auto target (85 bp) and long Deg target (294 bp) and to calculate the degradation index or degree of DNA degradation (the Auto/Deg ratio). In addition, the Y target was amplified to detect male DNA. PowerQuant reactions were duplicated, and average results were reported. QPCR reactions were carried out following the recommendations provided in the technical manual [32]. Detection of PCR inhibitors was possible through internal PCR control and IPC shift value. Reaction was carried out on the QuantStudio 5 Real-Time PCR system (Applied Biosystems, AB, Foster City, CA, USA) using Quant-Studio Design and Analysis Software 1. 5. 1.

### STR typing

For missing person identification, highly informative autosomal STR genetic profiles are required [17, 33]) for verification of kinship through comparison to the profiles of family reference persons. All the extracts obtained from teeth and petrous bones were amplified for autosomal STRs, and the number of STR loci that were successfully amplified was determined. In this manner, the utility of DNA extracted for genetic identification was verified because STR typing is a final step in genetic identification. STR genetic profiles were also used to confirm the genuineness of isolated DNA via comparison with the profiles in the elimination database. To confirm the endogenous DNA, genetic profiles obtained from teeth were compared to profiles obtained from petrous bones sampled from the same skeleton. If no match was found with the elimination database, the degradation indexes measured were high, the ENCs produced no profiles, and the genetic profiles acquired from tooth‒petrous bone pairs sampled from the same skeletons matched, then extraction of endogenous tooth and bone DNA was confirmed and there had been no modern DNA contamination.

Following the manufacturer’s guidelines [34], the Nexus MasterCycler (Eppendorf, Hamburg, Germany) was used for 30 cycles of PCR reaction utilizing the PowerPlex ESI 17 Fast System (Promega). PCR reaction was carried out with one ng of template DNA if the qPCR Auto target was higher than 0.058 ng DNA per μl, and the largest possible input volume (17.5 μl) of extract was used in extracts with quantities lower than 0.058 ng DNA per μl. An equal volume was utilized for ENC samples. In addition, every sample in the elimination database underwent autosomal STR typing. STR profiles were obtained using WEN Internal Lane Standard 500 (Promega), the SeqStudio Genetic Analyzer for HID (Thermo Fisher Scientific, TFS), GeneMapper ID-X Software v 1.6 (TFS), and SeqStudio Data Collection Software v 1.2.1 (TFS).

### Statistical analysis

Of interest was whether the quantity and quality of DNA obtained from petrous bones differ from those obtained from tooth cementum. Statistical analysis was performed on three parameters: the DNA yield (qPCR PowerQuant Auto target, expressed in ng DNA/µl of extract), degree of DNA degradation (qPCR PowerQuant Auto/Deg ratio), and success of STR typing (number of successfully amplified STR loci). All three parameters were compared between 60 petrous bones and 60 teeth. Because a quarter of the teeth did not generate genetic profiles (see Results, STR typing), additional statistical analysis was performed excluding 15 teeth that lacked genetic profiles.

Based on the research interest, the following research hypothesis was formed: there are differences in petrous bones and tooth cementum in DNA yield, degree of DNA degradation, and success of STR typing.

The Kolmogorov–Smirnov test (using Lilliefors significance correction) was employed to test the normality and homogeneity of variance. The research hypotheses were checked using 95% confidence intervals for means or medians, as recommended as a suitable measure for testing inter-group differences, specifically in medical studies [35–37], utilizing the program IBM SPSS Statistics for Windows, version 28.0 (Statistical Package for the Social Sciences Inc., Chicago, IL, USA). Because there is a relatively small sample size, the confidence intervals may have limited capacity to identify significant differences [37]. Thus, the formulated hypotheses were also tested using *p* values. Significance was set as *p* ≤ 0.05.

The database contained data acquired from 60 petrous bones and 60 teeth. There were two cases, both teeth, in which there were no data for either of the quantitative variables (Auto target and Auto/Deg ratio). Because there were not enough data, this sample was omitted from the database. There were also three teeth and one petrous bone from which the Auto target was acquired, whereas it was not possible to calculate the value for the Auto/Deg ratio because no Deg targets had been amplified. In the first phase, the following formula was used to calculate the Auto/Deg ratio for these cases: value(i) = Max(i) + *SD*(i), where (i) represents a specific skeletal element. Degradation ratio values that were missing were set to 413.88 (335.37 + 78.51) for petrous bones and 71.35 (57.54 + 13.81) for teeth. The values that were calculated were then saved in the database.

The Kolmogorov–Smirnov test indicated that the data do not show a normal distribution. Thus, non-parametric tests were performed, and medians were used for the confidence intervals.

## Results

### DNA quantification

The PowerQuant Auto target was used as a DNA quantity measurement and the Auto/Deg ratio as a DNA quality measurement (degradation index). The data obtained using the PowerQuant System (Promega) are shown in Supplementary Material (SM) 1. Auto, Deg, and Y target amplification results are expressed in ng DNA/µl of extract. Auto and Deg targets were not detected in two tooth samples, and only Deg target amplification was missing in three tooth samples and one petrous bone extract. Less than 0.5 pg of DNA per μl of extract—the lower limit of detection for reliable quantification results determined by the validation study for PowerQuant System [38]—was detected in seven tooth samples (see SM 1, labeled in blue). If the value of the IPC shift exceeds 0.3, it shows a possible presence of PCR inhibitors. In three petrous bones and one tooth sample, the IPC shift value exceeded 0.3, but it did not surpass 0.75 (see SM 1, labeled in red), and all four DNA extracts produced highly informative autosomal STR genetic profiles, indicating high purification efficiency of the EZ1 & EZ2 DNA Investigator kit (Qiagen), which utilizes magnetic bead technology. DNA degradation in tooth samples was lower than DNA degradation in petrous bone samples. In teeth, values for the degradation index ranged from 0.55 to 57.54, and in petrous bones from 3.75 to 335.37. In four samples (three teeth and one petrous bone), the degree of DNA degradation could not be determined because of an undetected PowerQuant Deg target (see SM 1, noted as “Undetermined”). In six out of 10 ENC samples (five processed together with teeth and five together with petrous bones), very small quantities of DNA were detected; however, no STR profiles were generated (see SM 1), showing no contamination issue.

### STR typing

All 60 petrous bones analyzed produced highly informative autosomal STR genetic profiles, with more than 11 loci amplified. According to the International Commission on Missing Persons (ICMP) submission criteria, genetic profiles consisting of at least 11 autosomal STRs are sufficiently informative for kinship verification [39]. Out of 60 tooth samples, 45 generated highly informative STR profiles, and in 15 samples STR genetic typing failed (see SM 1, column ESI-17 STRs, labeled in green), and for all of them low DNA quantity was measured with the qPCR PowerQuant kit (Promega). For the Črnomelj archaeological site five teeth did not produce genetic profiles, and for the Ljubljana archaeological site genetic typing failed in 10 tooth samples.

Profiles obtained from petrous bones matched the profiles from the teeth sampled from the same skeleton, confirming endogenous DNA. No elimination database match and clean negative controls also confirmed endogenous DNA. Moreover, the authenticity of DNA acquired from teeth and petrous bones was verified through high degradation indexes, especially in petrous bones.

### Statistical analysis

**Fig. 1:**
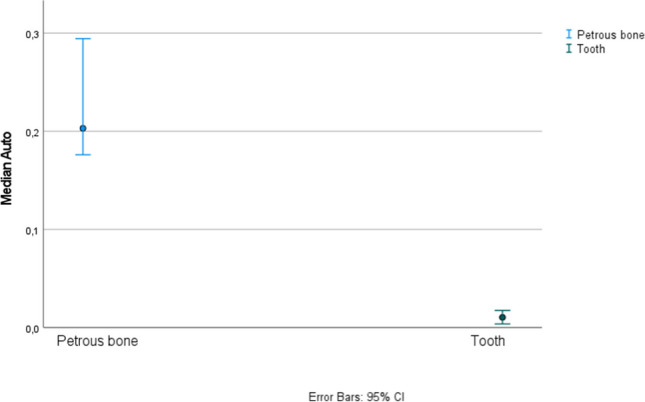
95% confidence intervals for medians for the Auto target (amount of DNA expressed in ng DNA/µl of extract) from petrous bones and tooth cementum

**Fig. 2:**
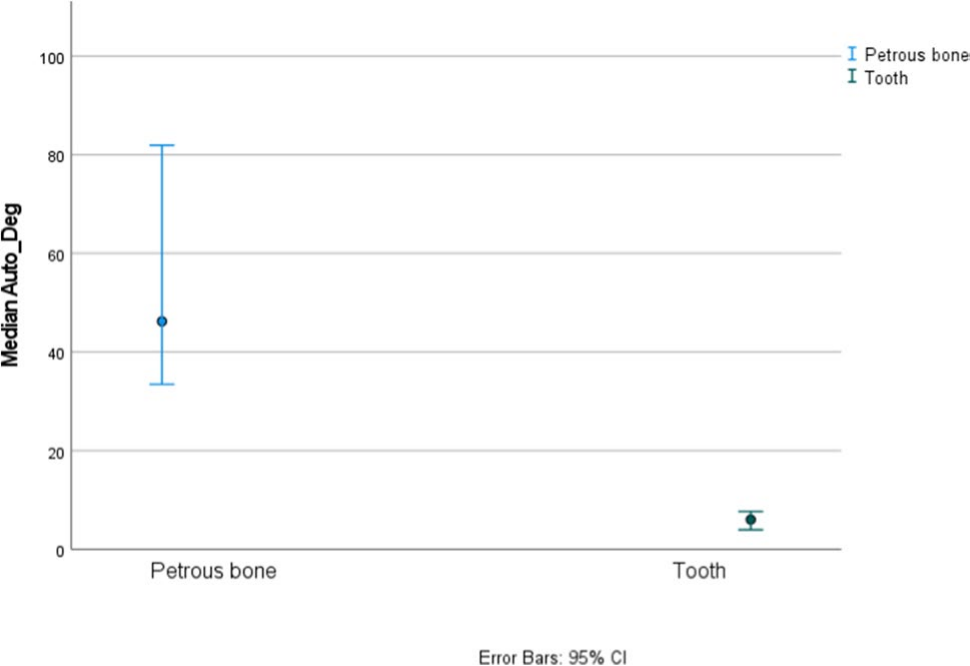
95% confidence intervals for medians for the Auto/Deg ratio from petrous bones and tooth cementum

**Fig. 3:**
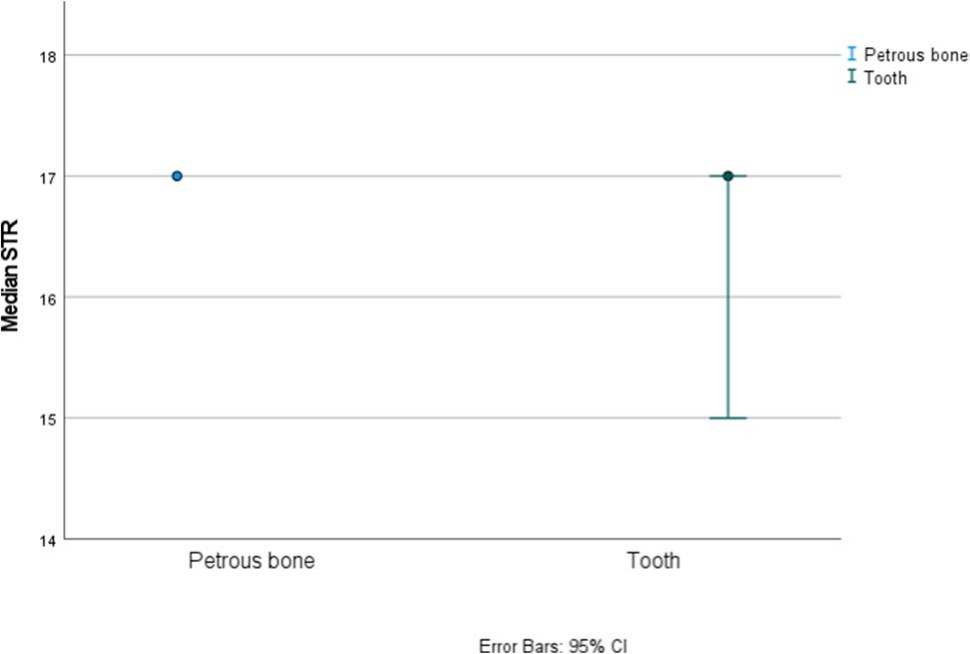
95% confidence intervals for medians for the STR typing success from petrous bones and tooth cementum considering all teeth analyzed

Statistical analysis was carried out to evaluate differences in DNA yield, degree of DNA degradation and autosomal STR amplification success between tooth cementum and petrous bones. All three parameters were statistically significantly higher in the petrous bone according to results of confidence intervals and an independent-sample median test (see Fig. 1 for the Auto target, Fig. 2 for the Auto/Deg ratio, and Fig. 3 for STR typing success, where the *p* value was 0.003, the petrous bone *Mdn* = 16.28, *SE* = 0.19, and the tooth *Mdn* = 12.53, *SE* = 0.91).

**Fig. 4:**
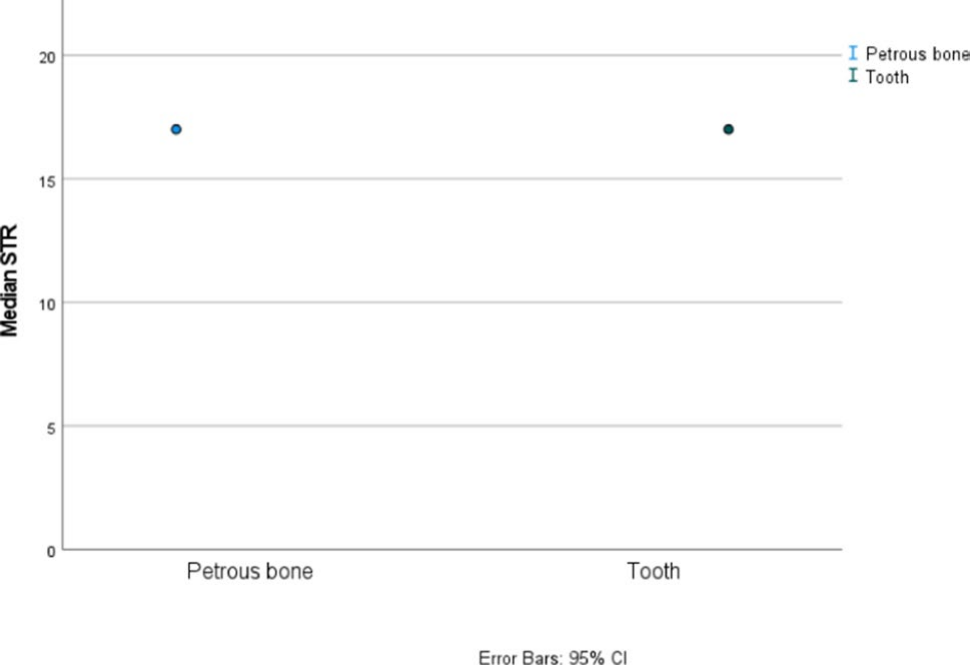
95% confidence intervals for medians for the STR typing success from petrous bones and tooth cementum excluding teeth lacking genetic profiles

Because a quarter of teeth did not generate genetic profiles, further statistical analysis was carried out excluding 15 teeth that lacked DNA profiles to simulate a more probable forensic outcome, in which teeth with better-preserved DNA can be expected. Namely, in typical forensic tooth samples the time of exposure to decay is much shorter than in ancient teeth analyzed, which ensure better-preserved tooth DNA. Excluding teeth lacking DNA profiles from statistical analysis, the DNA yield (Auto target) and DNA degradation (Auto/Deg ratio) were again significantly higher in petrous bones, but no significant difference was found in the success of STR typing (*p* = 0.482; petrous bone *Mdn* = 16.28, *SE* = 0.19, and tooth *Mdn* = 16.16, *SE* = 0.22) and confidence interval overlaps (see Fig. 4).

## Discussion

In ancient DNA analysis, the pars petrosa of the temporal bone, collection of which is destructive, is used as the most promising bone type for obtaining optimal DNA yield [5]. When destructive sampling is proposed to museums, they must balance an advance in scientific knowledge against losing priceless material that cannot be replaced [40]. Often, destructive sample collection is inappropriate for historical remains or for museum specimens because the petrous bone can also be used for other analyses [26, 41]. Austin et al. [40] emphasize its value for stable isotope analysis as a proxy or supplement for teeth when reconstructing diet during early life [42], significant morphological markers of population histories [43], and morphological data for sex and childhood disease [15]. Minimally invasive approaches might present an alternative solution to successfully recover DNA from skeletal remains [16]. The nondestructive extraction method from tooth cementum applied in this study is an example of a minimally invasive approach. The results of this study analyzing teeth and petrous bones collected from skeletons dated from the 13th to nineteenth century showed the high potential of tooth cementum for genetic analysis in 75% of skeletons. In the rest of the skeletons, teeth showed poorly preserved DNA. However, teeth from forensic cases are better preserved than teeth from archaeological skeletons because their exposure to harmful environmental conditions is shorter. Given better DNA preservation, a high success rate (greater than the 75% obtained for ancient teeth) is expected in teeth from forensic casework. The results of this study showed that, even if DNA yield was significantly higher in petrous bones, the same STR amplification success was attained in teeth when only teeth with preserved DNA were considered for comparison due to lower degradation of DNA obtained from tooth cementum. Consequently, when aged skeletal remains need to be genetically typed, tooth cementum of well-preserved teeth can be processed instead of petrous bones. Very similar results were obtained when much older ancient skeletal remains (Bronze Age, Iron Age, Viking period, and some skeletons dated to later historical times in Denmark) were investigated, and petrous bone and tooth cementum were recognized as an excellent source of DNA for ancient genetic analyses [15]. However, because of some badly preserved teeth from the Viking period, statistical analysis showed better performance of petrous bones than teeth. Omitting Viking-period skeletons, no differences were recognized between petrous bones and tooth cementum. Hansen et al. [15] confirmed a link between visual preservation and DNA preservation of teeth, and they concluded that well-preserved teeth perform as well as petrous bones [15].

Petrous bones and teeth are both characterized as the hardest tissues in the human body. However, this study showed higher DNA degradation in petrous bones than in tooth cementum. When destructive extraction methods are employed in obtaining DNA from petrous bones, there is a possibility of heat damage to DNA and, accordingly, liquid nitrogen was used to prevent overheating [27]. Because of higher degradation indexes in petrous bones, it is possible that DNA degradation occurred even if liquid nitrogen was used to cool bone samples and metal grinding jars before bone powdering. In addition to overheating, mechanical damage is possible during grinding. When using a nondestructive isolation method, teeth were not exposed to grinding, and no DNA damage occurred during DNA extraction. The difference in the extraction procedure (destructive for petrous bones and nondestructive for teeth) could explain higher DNA degradation in petrous bones. In addition to protecting tooth DNA against degradation, the nondestructive extraction method physically preserves the specimen, which is important for relatives when skeletal remains have to be returned to them after missing person identification due to ethical issues [44]. In addition, absence of grinding and consequently reduced manual handling makes tooth samples less prone to contamination. Indeed, minor contamination issues (some sporadic drop in alleles) were observed in a few petrous bone extracts but not in extracts obtained from teeth, indicating possible exposure of endogenous DNA to contamination during the grinding process. However, in comparison to bones, teeth are by their nature less prone to contamination than bones because they are less porous and they are protected by impermeable enamel [45].

## Conclusions

This study showed that well-preserved teeth are a good source of DNA and that tooth cementum can be used for genetic analysis. There are several advantages when using tooth cementum instead of petrous bones. First, a nondestructive extraction method can be applied to tooth cementum, which allows for physical preservation of teeth for morphological analysis. Second, no need for grinding shortens the extraction procedure and reduces the possibility of contamination. Third, no liquid nitrogen is needed for extraction of DNA from tooth cementum. Fourth, by omitting the grinding process, tooth DNA is less exposed to degradation. Applying simple and rapid nondestructive extraction from tooth cementum, teeth can be used as the first-choice biological specimen for identification of skeletonized missing persons in forensic casework. In cases of unsuccessful typing, proceeding with more time-consuming destructive extraction of DNA from the petrous bone is recommended.

This study offers valuable insights into potentially using tooth cementum as a reliable DNA source for investigating aged skeletal remains, highlights the importance of nondestructive extraction method for DNA analysis, and offers practical suggestions for forensic investigations.

## Supplementary Information

Below is the link to the electronic supplementary material.

Supplementary file1 (XLSX 77.2 KB)

## Data Availability

The authors declare that all the data are available.
